# Isolation of colonization-defective *Escherichia coli* mutants reveals critical requirement for fatty acids in bacterial colony formation

**DOI:** 10.1099/mic.0.000673

**Published:** 2018-07-20

**Authors:** Kazuki Nosho, Koji Yasuhara, Yuto Ikehata, Tomohiro Mii, Taichiro Ishige, Shunsuke Yajima, Makoto Hidaka, Tetsuhiro Ogawa, Haruhiko Masaki

**Affiliations:** ^1^​ Department of Biotechnology, The University of Tokyo, Yayoi, Bunkyo-ku, Tokyo, Japan; ^2^​ NODAI Genome Research Center, Tokyo University of Agriculture, Setagaya-ku, Tokyo, Japan; ^3^​ Department of Bioscience, Tokyo University of Agriculture, Setagaya-ku, Tokyo, Japan; ^†^​ Present address: Graduate School of Frontier Sciences, The University of Tokyo, 5-1-5 Kashiwanoha, Kashiwa, Chiba 277-8562, Japan.

**Keywords:** *Escherichia coli*, colony formation, fatty acid, *fabB*, c.f.u., MPN

## Abstract

Most bacterial cells in nature exhibit extremely low colony-forming activity, despite showing various signs of viability, impeding the isolation and utilization of many bacterial resources. However, the general causes responsible for this state of low colony formation are largely unknown. Because liquid cultivation typically yields more bacterial cell cultures than traditional solid cultivation, we hypothesized that colony formation requires one or more specific gene functions that are dispensable or less important for growth in liquid media. To verify our hypothesis and reveal the genetic background limiting colony formation among bacteria in nature, we isolated *Escherichia coli* mutants that had decreased frequencies of colony formation but could grow in liquid medium from a temperature-sensitive mutant collection. Mutations were identified in *fabB*, which is essential for the synthesis of long unsaturated fatty acids. We then constructed a *fabB* deletion mutant in a wild-type background. Detailed behavioural analysis of the mutant revealed that under fatty acid-limited conditions, colony formation on solid media was more sensitively and seriously impaired than growth in liquid media. Furthermore, growth under partial inhibition of fatty acid synthesis with cerulenin or triclosan brought about similar phenotypes, not only in *E. coli* but also in *Bacillus subtilis* and *Corynebacterium glutamicum*. These results indicate that fatty acids have a critical importance in colony formation and that depletion of fatty acids in the environment partly accounts for the low frequency of bacterial colony formation.

## Introduction

Micro-organisms, particularly those in the domain *Bacteria*, are the dominant cellular life forms on Earth, with enormous diversity in genes and bioactive metabolites. For over a century, microbiologists have used a pure culture technique based on colony formation to isolate novel micro-organisms. However, numerous studies have reported that only a tiny fraction of bacterial cells living in nature can form colonies on agar plates [1, 2]. This low frequency of colony formation imposes severe limitations on our knowledge and utilization of enormous bacterial resources for biotechnological and medical applications.

To isolate novel bacteria, various improvements in culturing conditions have been developed, e.g. diluting nutrients for culturing oligotrophs [3–7], removing reactive oxygen species [8–10], using alternative gelling agents to avoid agar-associated growth inhibition [10–13], co-culturing with helper bacteria [14–17] and adding growth-promoting factors [4, 18, 19]. Furthermore, recent advances in microdevices, combined with previous knowledge, have enabled high-throughput culturing techniques to be developed [20–24]. These methods have resolved individual problems related to culture conditions. However, the great majority of bacteria have still not been cultivated [25–30]. There is no persuasive general explanation regarding what makes bacteria in nature so difficult to culture on solid media.

In 1979, Kogure *et al*. invented a reliable method for microscopically counting viable cells in a marine sample [31]. Marine bacteria were cultivated in sea water supplemented with a trace amount of nutrients and nalidixic acid. Under this condition, viable cells elongated, but stopped dividing because of the inactivation of DNA gyrase by nalidixic acid, resulting in filamentous shapes. They enumerated these filamentous cells as viable cells with the potential to proliferate. Based on this new viable cell count index, they proposed a ‘direct count of viable bacterial cells (DVC)’, and found that the colony-forming unit (c.f.u.), a commonly used parameter for quantifying viable cells, was several orders of magnitude smaller than DVC, suggesting that a great discrepancy between colony formation on agar plates and cell division exists.

Because DVC indicates the number of cells capable of cell division in liquid culture, liquid cultivation may yield many more bacterial cell cultures than traditional plating methods. Indeed, numerous studies have reported that liquid cultivation typically yields higher cultivation efficiencies than solid cultivation, which has sometimes enabled the isolation of novel bacteria from various natural habitats without colony formation [5–7, 32–34]. These results suggest that there are limitations to colony formation, and that colony formation is a specific biological event that is distinguishable from growth in liquid culture. We hypothesized that colony formation requires one or more specific gene functions, which are dispensable or less important for growth in liquid media. The identification and analysis of such genes may explain the growth differences between solid and liquid cultures, revealing the main cause of the low colony-forming activity of bacteria in the natural environment. To identify the genes required for colony formation, the most straightforward approach is to isolate the mutants that have lost their colony-forming ability. Therefore, in this study, we first focused on obtaining *Escherichia coli* mutants that could not form colonies but grew in liquid media.

In this study, we attempted to screen colonization-defective mutants that retained culturability in liquid media from a temperature-sensitive *E. coli* mutant collection [35]. Temperature-sensitive mutants are those that do not form colonies at non-permissive temperatures; however, their corresponding culturability in liquid culture has not been examined comprehensively. Therefore, we expected that the temperature-sensitive mutant collection contains objective *E. coli* mutants that cannot form colonies but grow in liquid culture at high temperatures, which might explain the gap in culturability between liquid and solid media. We successfully obtained colonization-defective *E. coli* mutants that grew in liquid media. Detailed characterization of these mutants revealed that colony formation requires more fatty acids on solid media compared to the requirements in liquid media. Moreover, we experimentally demonstrated that these results can be extended to wild-type *E. coli* and possibly other bacteria, indicating an important role for fatty acids in bacterial colony formation.

## Methods

### Bacterial strains and culture conditions

The *E. coli* K-12 wild-type strain used in this study was MG1655. An *E. coli* temperature-sensitive (ts) mutant collection derived from PA3092 [35] and an F^+^ strain, PA200, with a plasmid library of *E. coli* open reading frames (ORFs) [36], were gifts from Dr Akiko Nishimura (formerly of the National Institute of Genetics, Japan). The *Bacillus subtilis* 168 and *Corynebacterium glutamicum* ATCC13032 were provided by Dr Hirofumi Yoshikawa (Tokyo University of Agriculture) and Dr Saori Kosono (The University of Tokyo), respectively. The *Escherichia coli* was cultivated in l-broth containing 1.0 % Bacto tryptone (BD Biosciences, Franklin Lakes, NJ, USA), 0.5 % Bacto yeast extract and 0.5 % NaCl, or M9 minimal medium containing 0.6 % Na_2_HPO_4_, 0.3 % KH_2_PO_4_, 0.1 % NH_4_Cl, 0.1 % NaCl, 1 mM MgSO_4_ and 0.1 mM CaCl_2_, with the addition of 0.5 % casamino acids and 0.4 % d-glucose or 0.4 % d-galactose, which are referred to as M9CAGlc and M9CAGal, respectively. For PA3092, 40 µg ml^−1^ thymine was added to the l-broth. The *Bacillus subtilis* was cultivated in SPMM medium containing 1.4 % K_2_HPO_4_, 0.6 % KH_2_PO_4_, 0.2 % (NH_4_)_2_SO_4_, 0.1 % sodium citrate dihydrate, 0.02 % MgSO_4_ and 50 µg ml^−1^ tryptophan, with the addition of 0.5 % casamino acids and 0.5 % d-glucose, which is referred to as SPMMCAGlc. The *Corynebacterium glutamicum* was cultivated in CM2B medium containing 1.0 % HiPolypepton (Wako Pure Chemical Industries, Ltd, Osaka, Japan), 1.0 % Bacto yeast extract and 0.5 % NaCl. To prepare the 1.5 % solid medium, agar powder (Kokusan Chemical Co., Ltd, Tokyo, Japan) was used for the screening of ts mutants, Bacto agar (BD Biosciences) was used for c.f.u. and MPN (most probable number) measurements, and Agarose L03 (Takara Bio, Inc., Shiga, Japan) was used for the other experiments.

### Preparation of chemical reagents

Oleic acid (Wako Pure Chemical Industries, Ltd) was dissolved in 1 % Triton X-100 (MP Biomedicals, Inc., Santa Ana, CA, USA) at 1, 7.5, 100 or 2000 µg ml^−1^ before addition to the culture medium. The final concentrations of these solutions in the medium were 0.01, 0.075, 1.0 and 20 µg ml^−1^, respectively. Palmitoleic acid (Tokyo Chemical Industry Co., Ltd, Tokyo, Japan) or *cis*-vaccenic acid (Funakoshi Co., Ltd, Tokyo, Japan) was dissolved in 1 % Triton X-100 at 2000 µg ml^−1^, and then diluted 100-fold with medium, giving a final concentration of 20 µg ml^−1^. l-Arabinose (Wako Pure Chemical Industries, Ltd) was dissolved in sterile water at 200 mg ml^−1^ and diluted with medium, giving final concentrations of 50, 125, 250, 500, 1000, 2000 and 4000 µg ml^−1^, respectively. Cerulenin (Wako Pure Chemical Industries, Ltd) was dissolved in 50 % ethanol at 2, 4, 6 or 8 mg ml^−1^ and then diluted with medium, giving final concentrations of 20, 40, 60 and 80 µg ml^−1^, respectively. For *E. coli*, triclosan (Wako Pure Chemical Industries, Ltd) was dissolved in 50 % ethanol at 5, 10, 15 or 20 µg ml^−1^ and then diluted with medium, giving final concentrations of 50, 100, 150 and 200 ng ml^−1^, respectively. For *B. subtilis*, triclosan (Wako Pure Chemical Industries, Ltd) was dissolved in 50 % ethanol at 25, 50, 75, 100, 125 or 150 µg ml^−1^ and then diluted with medium, giving final concentrations of 250, 500, 750, 1000, 1250 and 1500 ng ml^−1^, respectively. For *C. glutamicum*, triclosan (Wako Pure Chemical Industries, Ltd) was dissolved in 50 % ethanol at 100, 200, 300 or 400 µg ml^−1^ and then diluted with medium, giving final concentrations of 1, 2, 3 and 4 µg ml^−1^, respectively. Ampicillin sodium (Wako Pure Chemical Industries, Ltd) was dissolved in sterile water at 2, 4, and 6 mg ml^−1^ and then diluted with medium, giving final concentrations of 20, 40 and 60 µg ml^−1^, respectively.

### Screening of colonization-defective ts mutants

Colonization-defective ts mutants were obtained as follows. The actual temperature of the sample plates was determined with a temperature logger (Thermochron SL; KN Laboratories, Osaka, Japan). A total of 1432 clones from the ts collection were grown overnight at 30 °C in 150 µl l-broth in microtitre plates. Each cell suspension was diluted 7500-fold with 150 µl l-broth, and aliquots of approximately 2 µl were inoculated using a Vaccu-Pette/96 (Bel-Art Sciencewear, Wayne, NJ, USA) into both l-agar plates and 150 µl of l-broth in microtitre plates. After incubation without shaking for 2 days at 41 °C, only one clone grew in the l-broth, while it did not grow on l-agar at 41 °C. To confirm the phenotype of this candidate, after overnight culture at 30 °C, the cells were serially diluted 30-fold with 150 µl of l-broth, and a 2-µl aliquot of each diluted solution was then inoculated onto l-agar and into l-broth. After incubation for 2 days at 41 °C, the clone exhibited a threefold discrepancy between colony formation on l-agar and turbidity formation in l-broth.

The ts mutation was mapped by complementation after the conjugative transfer of a plasmid library carrying an *E. coli* ORF set [36]. The temperature sensitivity of the growth on l-agar at 41 °C was complemented, i.e. colonies were only formed by a plasmid carrying *fabB*. PCR amplification and sequencing of this *fabB*^ts^ gene (*fabB-1*) revealed a C653-to-T transition in the 1221 bp ORF, corresponding to a change of Ala218 to Val in the FabB protein.

To confirm the reproducibility of the *fabB* mutant isolation, we screened an additional 1167 clones from the same ts collection, although this selection was performed at a more moderate temperature of 39 °C. After overnight pre-culture at 30 °C, each clone was serially diluted 30-fold in 150 µl l-broth, and 2-µl aliquots were then inoculated into both l-agar and l-broth. After incubation for 2 days at 39 °C, the colony formation on solid medium and the turbidity in liquid medium were compared. Three clones were isolated as having defects in colony formation. Mapping and sequencing of the three genes responsible for ts revealed that they included the second *fabB^ts^* gene (*fabB-2*) with a single mutation of G95 to A, corresponding to a change of Gly32 to Glu in FabB.

### Construction of a Δ*fabB* strain and mini-F plasmid carrying *fabB.*

The *fabB* ORF of *E. coli* MG1655 was replaced with the ORF of the Tn*5*-derived kanamycin phosphotransferase gene by homologous recombination using a pRed/ET Recombination system (Gene Bridges GmbH, Heidelberg, Germany), which yielded the *fabB*-deletion strain ∆*fabB*. Based on previous reports of *fabB* mutants, ∆*fabB* was constructed in the presence of 200 µg ml^−1^ oleic acid in the medium [37, 38]. The *fabB* gene accompanied by its 41 bp 5′ untranslated region was inserted into the *Nhe*I site of the mini-F plasmid pKLJ12 [39], yielding the recombinant plasmid pKLJ12-*fabB*, in which *fabB* was under the control of the *araBAD* promoter.

### c.f.u. and MPN measurements

Culturability on solid medium and in liquid medium were compared by determining c.f.u. and MPN, respectively. When determining the c.f.u., we did not use a glass spreader for the plating to avoid sample loss and the accompanying experimental error. Instead, cells were inoculated as follows. Bacterial cell suspensions were diluted with saline, and a 100 µl aliquot of each suspension and 650 µl of sterile water were added to a plate and spread on the entire surface by tilting the plate. The sterile water was added so that cells would spread to a sufficient extent over the entire surface of the plate. The plates were sealed with Parafilm and incubated at 30, 37 or 39 °C. c.f.u. values were expressed as colony-forming cell numbers per millilitre of the original culture. MPN was determined as follows. Bacterial cell suspensions were diluted appropriately with liquid medium, and 150 µl of each suspension was then dispensed into a 96-well microtitre plate. The plate, covered with a lid, was sealed with Parafilm and incubated at 30, 37 or 39 °C without shaking. The number of turbid wells per 96 wells was determined; the MPN was then calculated according to the Poisson distribution [40] and expressed as the number of culturable cells per millilitre of the original culture. The results were determined after changes in the number of colonies and the turbidity converged, as colony formation and turbidity formation are generally delayed under conditions where growth is hampered, e.g. after incubation for 4 days in the case of ts mutants, although most colonies and turbidity became visible within 1 day.

### Comparison of proliferation in solid and liquid cultures

To determine the number of cells capable of proliferating in solid and liquid media, strains MG1655 and ∆*fabB* were cultured overnight in M9CAGlc medium containing 20 µg ml^−1^ oleic acid. The cells were washed with saline, and 100 µl of each diluted solution was then inoculated onto several solid medium plates and into liquid medium, followed by incubation at 37 °C; the experimental media contained corresponding concentrations of oleic acid. At each sampling time, all cells were harvested from each plate with sterile water, and an appropriate volume was then spread on an l-agar plate containing 20 µg ml^−1^ oleic acid to count the proliferating cells per plate, as illustrated in Fig. S1 (available in the online version of this article). Similarly, an aliquot of the liquid medium was harvested and spread to count the number of proliferating cells per millilitre of liquid medium.

### Evaluation of the status of *E. coli* cells under stressed conditions

*Escherichia coli* MG1655 cells grown to OD_660_=0.8 were washed twice with saline, resuspended in sterile water to give a cell density of approximately 10^6^ cells ml^−1^, and then incubated at 4 °C. The total cell count was determined with a Multisizer 3 Coulter Counter (aperture diameter: 30 µm; Beckman Coulter, Inc., Brea, CA, USA). The viable cell count was determined with a LIVE/DEAD *Bac*Light Bacterial Viability kit (Molecular Probes, Inc., Eugene, OR, USA) according to the manufacturer’s recommendations. c.f.u. was determined with l-agarose or M9CAGlc-agarose plates with or without 20 µg ml^−1^ oleic acid after cultivation for 3 days at 25 °C.

### Cultivation of soil bacteria

Soil was sampled from the forest floor at a depth of approximately 20–30 cm at the University of Tokyo Tanashi Forest, Nishi Tokyo, Tokyo, Japan. The soil was divided into 1 g portions and stored at −80 °C. One gram of soil was suspended in 10 ml phosphate-buffered saline (PBS; 137 mM NaCl, 27 mM KCl, 8 mM Na_2_PO_4_ and 1.5 mM KH_2_PO_4_), vortexed and sonicated for 1 min. Soil particles were removed by paper filtration, and the supernatant was spread onto plates. For cultivation, 1/10 strength l-broth solidified with 1.5 % Agarose L03 (Takara Bio, Inc.) was used. A mixture of palmitoleic acid (Tokyo Chemical Industry Co., Ltd) and oleic acid (Wako Pure Chemical Industries, Ltd), dissolved in 1 % Triton X-100 (MP Biomedicals) at 2000 µg ml^−1^ each, was added and diluted 100-fold in medium as appropriate. Glycerol (Kokusan Chemical Co., Ltd) was added to the medium at a final concentration of 100 µg ml^−1^ as appropriate. After incubation for 10 days at 25 °C, the c.f.u. g^−1^ soil was calculated from the number of visible colonies formed on the solid medium.

## Results

### Screening of colonization-defective *E. coli* mutants

We first attempted to isolate colonization-defective mutants that only grew in liquid culture from a transposon-mutagenized library of *E. coli* using replica plating. However, all clones that grew in liquid medium formed colonies, suggesting that the gene(s) of interest were essential or that the mutants, if any, were leaky, with only decreased rather than eliminated colony-forming frequencies. We then attempted to obtain mutants with decreased colony-forming frequencies from a collection of ts *E. coli* strains [35]. These ts mutants did not form colonies at non-permissive temperatures, but their corresponding culturability in liquid medium has not been examined previously. Thus, we screened for mutants that could grow in liquid medium at non-permissive temperatures (see the Methods section).

Among the 1432 ts strains in the collection, only 1 clone capable of growing in liquid medium at 41 °C was identified, which is referred to as clone 1. Screening of the other 1167 ts strains at a more moderate temperature of 39 °C revealed 3 clones capable of growing in liquid medium, which are referred to as clones 2–4. After mapping [36] and sequencing the genes responsible for the ts mutations, clones 1 and 2 were found to have different single mutations in the same gene, *fabB*, which encodes 3-oxoacyl-(acyl carrier protein) synthase I, a key enzyme involved in the synthesis of long unsaturated fatty acids [37, 38, 41]. Hereafter, the two *fabB*^ts^ mutants, clone 1 and clone 2, are referred to as *fabB-1* and *fabB-2*, respectively (see the Methods section for a detailed description of the mutations). We are currently characterizing the genes responsible for ts and their effects for clones 3 and 4, which proved to have mutations on different genes related to folic acid metabolism.

### Characterization of *fabB* mutants

**Fig. 1.**
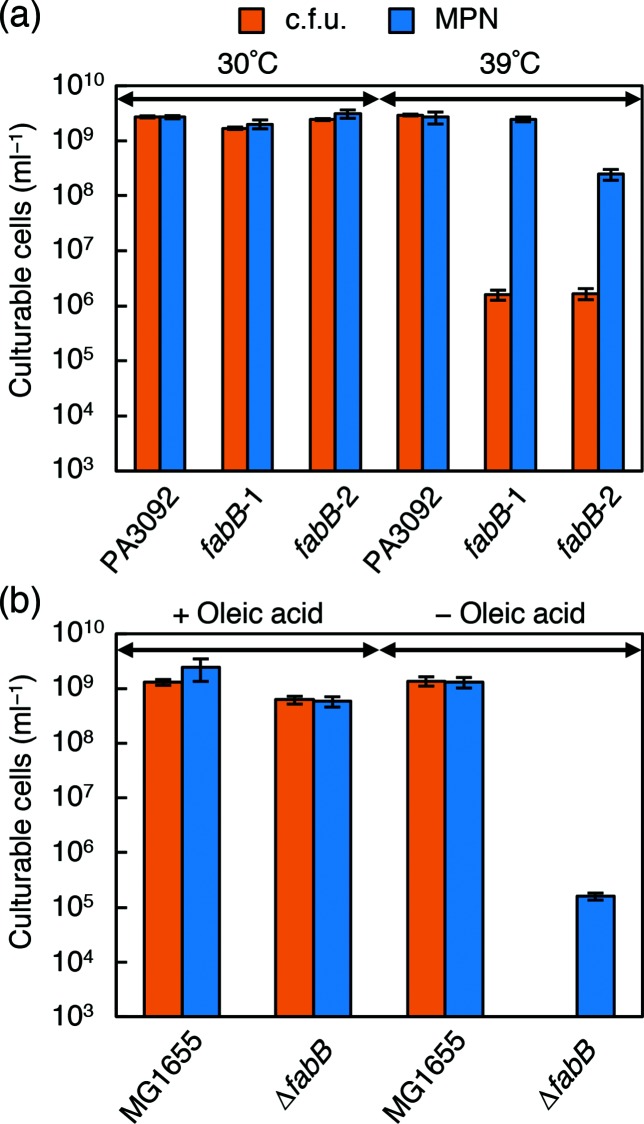
Comparison of culturability in solid and liquid media of *E. coli fabB* mutants. (a) The c.f.u. and MPN of PA3092 and its ts mutants (*fabB-1* and *fabB-2*) were determined in l-agar and liquid l-broth, respectively, after incubation for 4 days at 30 °C or 39 °C. (b) The c.f.u. and MPN of strains MG1655 and Δ*fabB* were determined after incubation for 4 days at 37 °C in l-agar and liquid l-broth, respectively, with or without 20 µg ml^−1^ oleic acid. Each experiment was performed in quadruplicate, and the error bars indicate standard deviations.

We compared the culturability of these mutant strains on solid media, quantified as c.f.u., with culturability in liquid media, quantified as MPN. Here, we used l-agar (l-broth-based agar) and liquid l-broth. At 39 °C the c.f.u. value of these mutants was approximately 1000-fold (*fabB-1*) or 100-fold (*fabB-2*) lower than the MPN value; in contrast, no difference was observed at 30 °C or at either temperature for the parental strain (Fig. 1a). These results suggest that *fabB* function is more important for colony formation than for growth in liquid culture.

Each ts clone, however, contained many unidentified background mutations in the genome resulting from heavy mutagenesis by methylnitronitrosoguanidine [35]. To evaluate the effects of *fabB* mutation alone, a deletion mutant, ∆*fabB*, was constructed from the wild-type strain MG1655 using medium containing oleic acid to complement the *fabB* defect [37, 38]. When the c.f.u. and MPN were compared in the presence of 20 µg ml^−1^ oleic acid, strain ∆*fabB* grew normally on l-agar and in liquid l-broth, similarly to the parental strain (Fig. 1b). In contrast, without oleic acid supplementation, strain ∆*fabB* did not form colonies on l-agar but grew in liquid l-broth, albeit at a low cell density. Although the *fabB* deletion mutation also affected growth in liquid culture to a greater extent than the ts mutations, the MPN was still at least 100-fold higher than the c.f.u. in strain ∆*fabB* (Fig. 1b). This was the expected result and supported the importance of the fatty acid supply for colony formation.

Palmitoleic acid (16 : 1 ω7c) and *cis*-vaccenic acid (18 : 1 ω7c), both abundant unsaturated fatty acids in *E. coli*, were as effective as oleic acid (18 : 1 ω9c) in supporting the growth of strain ∆*fabB* (see Fig. S2). We did not determine the effect of stearic or palmitic acids because of the low solubility of these saturated fatty acids, even with the use of 100 µg ml^−1^ Triton X-100 as a solubilizer.

### Proliferation of strain ∆*fabB* in solid or liquid culture

**Fig. 2.**
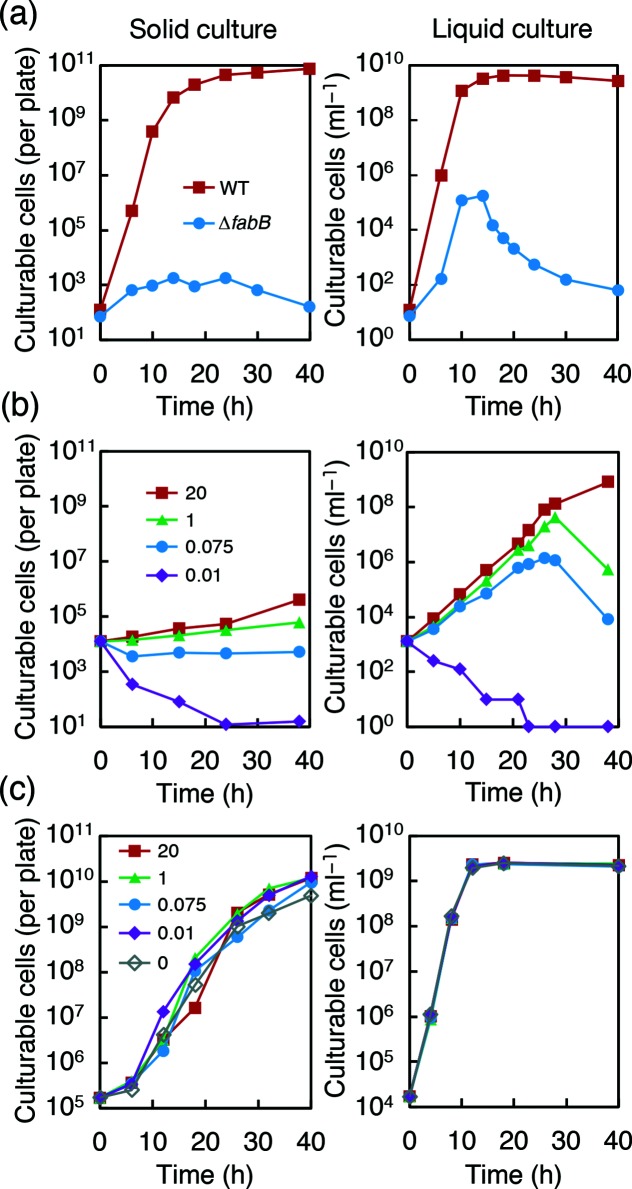
Cell proliferation of strains MG1655 and ∆*fabB* in solid (left) and liquid (right) cultures. (a) MG1655 and ∆*fabB* were inoculated onto l-agarose plates (left) and into l-broth medium (right), followed by incubation at 37 °C. Cells were periodically recovered from each plate or l-broth culture and spread on l-agar plates containing 20 µg ml^−1^ oleic acid to count colony-forming cells (see also the Methods section and Fig. S1). (b and c) Proliferation of strains ∆*fabB* (b) and MG1655 (c) was compared between solid (left) and liquid (right) cultures in M9CAGlc-based medium containing 0.01, 0.075, 1.0 or 20 µg ml^−1^ oleic acid. As in (a), cells were inoculated onto M9CAGlc-agarose plates and into M9CAGlc liquid medium. Cells were periodically recovered from each plate or medium for the counting of colony-forming cells.

The experiments described above did not exclude the possibility that ∆*fabB* cells proliferated slowly, forming invisible microcolonies on the plates. To evaluate the actual proliferation of strain ∆*fabB* in solid or liquid culture, we harvested cells from solid and liquid media after various incubation times and counted the cells by plating them with 20 µg ml^−1^ oleic acid (Fig. 2a; see also Fig. S1). To avoid possible growth inhibition by agar [10], we replaced the agar in the plates with purified agarose. We confirmed that the ∆*fabB* cells could not proliferate well on l-broth-based agarose (l-agarose), but proliferated in liquid l-broth, reaching more than 10^5^ cells ml^−1^ without fatty acid supplementation (Fig. 2a). These data indicate that impairment of fatty acid synthesis affects growth on solid medium to a greater extent than it does in liquid medium.

These results raised the question of how strain ∆*fabB* could proliferate to the extent that it did in liquid l-broth, as *fabB* is an essential gene [42]. Trace amounts of fatty acids, likely in the yeast extract in l-broth, may have partially compensated for the loss of FabB. However, fatty acid requirements may differ between solid and liquid cultures. To examine the correlation between the concentration of oleic acid and proliferation of ∆*fabB* cells precisely, we replaced l-broth with modified M9 medium containing casamino acids and glucose (M9CAGlc) and added specific amounts of oleic acid (Fig. 2b, c).

In the presence of oleic acid at all tested concentrations above 0.01 µg ml^−1^, the number of ∆*fabB* cells in the liquid culture exceeded 10^6^ cells ml^−1^, and the concentration of oleic acid did not substantially affect the growth rates of cells during the early stages. In contrast, the growth rates in solid culture were greatly reduced and significantly lower than those in liquid culture. Particularly, 0.075 µg ml^−1^ oleic acid was sufficient for transient growth in liquid culture, while no growth was observed on solid culture (Fig. 2b). The growth peak in the liquid culture suggests the exhaustion of the oleic acid supply. During the subsequent decrease in c.f.u., the viable cell count remained high, as estimated by LIVE/DEAD BacLight staining (see Fig. S3). A similar growth peak was reported for *E. coli* mutants that are auxotrophic for unsaturated fatty acid [43, 44].

### Effect of decreased *fabB* expression on colony formation

In addition to the two *fabB* ts mutants at non-permissive temperatures in l-broth (Fig. 1a), the ∆*fabB* deletion mutant in l-broth (Figs 1b and 2a) and in M9CAGlc-medium supplemented with a trace amount of oleic acid (Fig. 2b) exhibited growth differences between solid and liquid cultures. Based on analysis of the characteristics of these *fabB* mutants, we concluded that a limited supply of fatty acids increases the sensitivity of colony formation on solid media and seriously impairs growth compared to that in liquid media.

We were further interested in whether the characteristics of colonization-defective mutants could explain the behaviours of bacteria in general, at least for wild-type *E. coli*, under fatty acid depletion conditions.

**Fig. 3.**
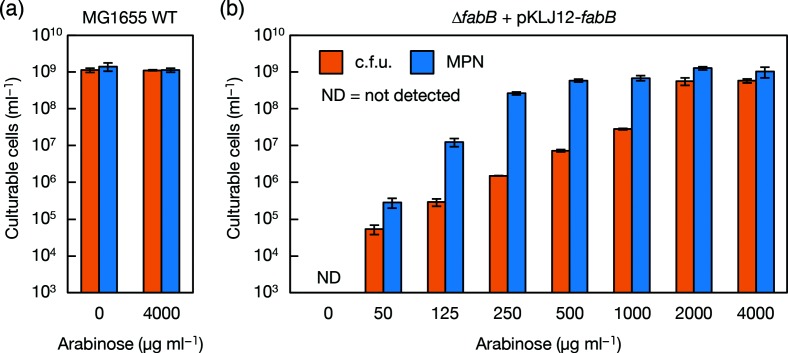
Comparison of culturability in solid and liquid media with reduced expression of *fabB*. The c.f.u. and MPN of (a) MG1655 and (b) MG1655Δ*fabB* carrying a miniF plasmid, pKLJ12-*fabB*, were determined after incubation at 37 °C for 6 days in M9CAGal-based solid and liquid media containing various concentrations of arabinose as indicated. Each experiment was performed in triplicate, and the error bars indicate standard deviations.

The wild-type strain showed no dependence on oleic acid in either liquid or solid cultures (Fig. 2a, c), reflecting the fact that the synthesis level was sufficient for growth under laboratory conditions without the addition of exogenous components. We then designed an *E. coli* strain with properties that were intermediate between those of the *fabB* deletion mutant and the wild-type. A miniF plasmid, pKLJ12 [39], harbouring *fabB* under control of the arabinose promoter was introduced into the ∆*fabB* strain. The c.f.u. and MPN of this strain, ∆*fabB* +pKLJ12-*fabB*, were compared in the presence of various concentrations of arabinose (Fig. 3). When 4000 µg ml^−1^ of arabinose was added, the c.f.u. and MPN values were nearly the same and comparable to those of the wild-type strain (Fig. 3a, b). Sufficient expression of *fabB* in the ∆*fabB* host strain complemented the deletion of genomic *fabB*. As the concentration of arabinose was decreased, the c.f.u. values became lower than the MPN values; at 250 µg ml^−1^ arabinose, the c.f.u. was more than 100-fold lower than the MPN (Fig. 3b). These results indicate that the reduction of *fabB* expression alone in the wild-type *E. coli* reduced c.f.u. more sensitively than MPN, inducing a phenotype that was similar to that of the *fabB* mutants.

### Effect of chemical inhibition of fatty acid synthesis with cerulenin

**Fig. 4.**
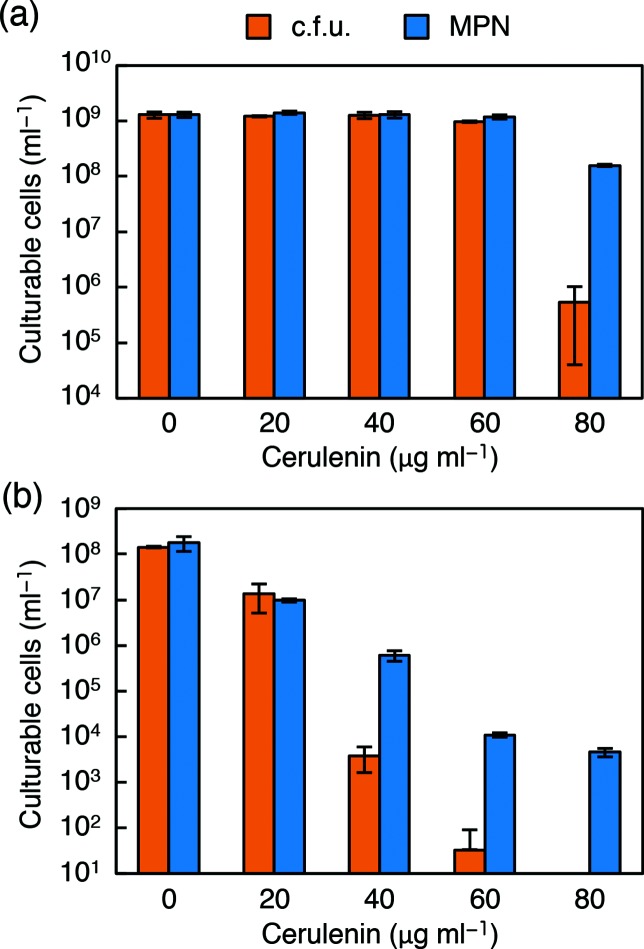
Comparison of culturability in solid and liquid media with reduced activity of 3-oxoacyl-ACP synthase of *E. coli* and *B. subtilis*. (a) The c.f.u. and MPN of *E. coli* MG1655 were determined after incubation at 37 °C for 3 days in M9CAGlc-based solid and liquid media containing various concentrations of cerulenin as indicated. (b) The c.f.u. and MPN of *B. subtilis* 168 were determined after incubation at 37 °C for 8 days in SPMMCAGlc-based solid and liquid media containing various concentrations of cerulenin. Each experiment was performed in triplicate, and the error bars indicate standard deviations.

To further determine the status of wild-type cells under fatty acid depletion conditions, we examined the effects of cerulenin. This antibiotic specifically inhibits FabB, and to a lesser extent its isoform FabF, in *E. coli* [45, 46], and inhibits fatty acid synthesis in a wide range of micro-organisms [47]. In the presence of a sublethal concentration of cerulenin (80 µg ml^−1^), the c.f.u. of wild-type *E. coli* was more than 100-fold lower than the MPN (Fig. 4a), indicating a relative reduction in colony-forming activity in response to the chemical inhibition of FabB, and possibly FabF, in fatty acid synthesis.

We conducted a similar experiment with *B. subtilis. B. subtilis* does not contain FabB. Its isoform FabF acts solely as the elongating 3-oxoacyl-ACP synthase and is known to be the target of cerulenin in this organism [48, 49]. *B. subtilis* was more susceptible to chemical inhibition with cerulenin than *E. coli* (Fig. 4b). In particular, colony formation was completely impaired at 80 µg ml^−1^, while modest growth was observed in liquid culture.

### Effect of chemical inhibition of fatty acid synthesis with triclosan

**Fig. 5.**
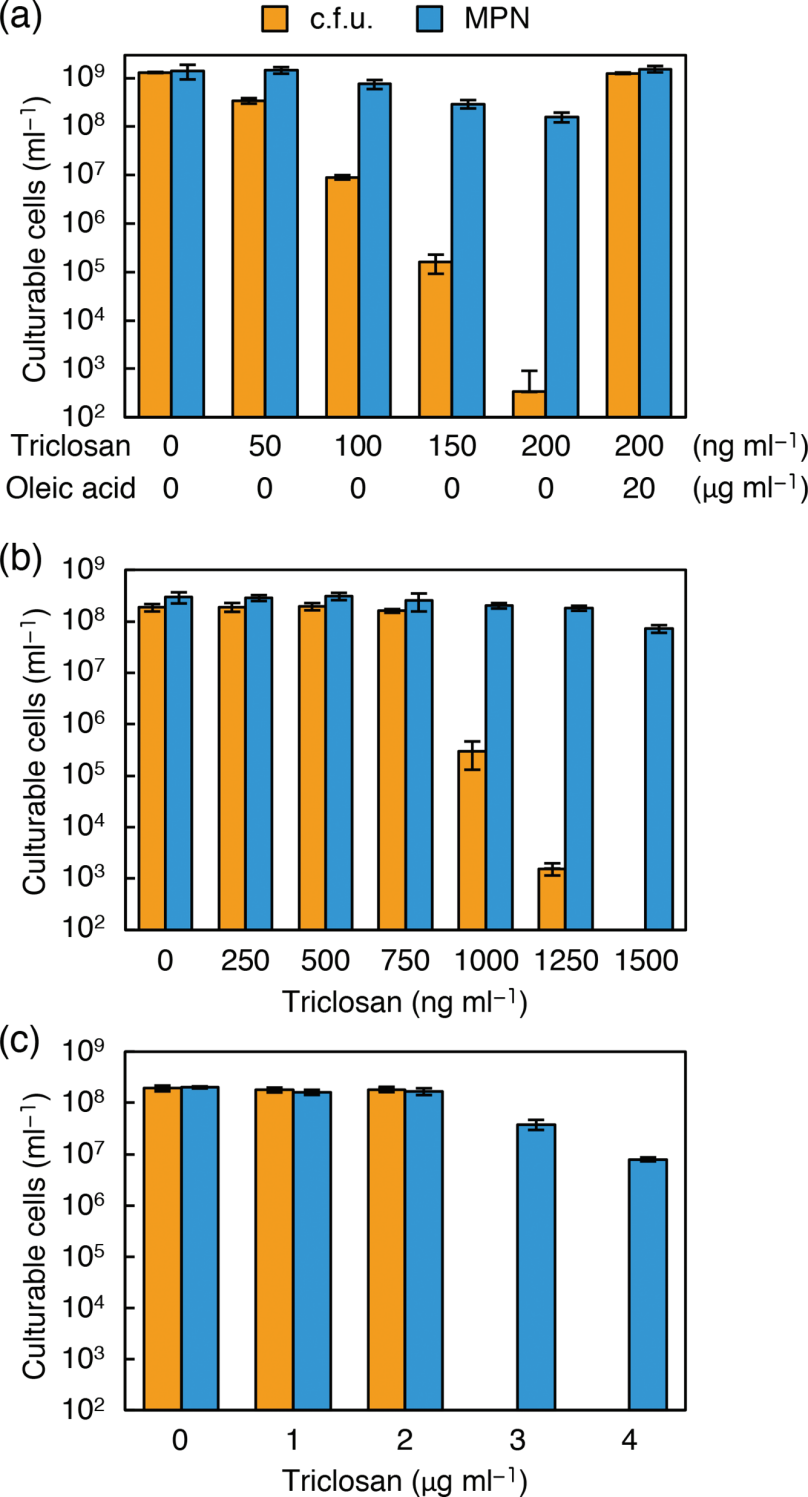
Comparison of culturability in solid and liquid media with reduced activity of enoyl-ACP reductase of *E. coli*, *B. subtilis* and *C. glutamicum.* (a) The c.f.u. and MPN of *E. coli* MG1655 were determined after incubation at 37 °C for 4 days in M9CAGlc-based solid and liquid media containing various concentrations of triclosan as indicated. (b) The c.f.u. and MPN of *B. subtilis* 186 were determined after incubation at 37 °C for 4 days in SPMMCAGlc-based solid and liquid media containing various concentrations of triclosan. (c) The c.f.u. and MPN of *C. glutamicum* ATCC13032 were determined after incubation at 30 °C for 4 days in CM2B-based solid and liquid media containing various concentrations of triclosan. Each experiment was performed in triplicate, and the error bars indicate standard deviations.

We next examined the effect of triclosan, which inhibits enoyl-ACP reductase I (FabI) in *E. coli* [50] and has been widely used as an antibacterial additive reagent. The relative reduction in c.f.u. compared to MPN by triclosan was more prominent than that by cerulenin in *E. coli* (Fig. 5a). In this experiment, exogenous supplementation with fatty acids completely overcame this large reduction of c.f.u., confirming that the deficit of fatty acids was responsible for this phenotype.

As a well-known bacteria that are distantly related to *E. coli*, *B. subtilis* and *C. glutamicum* were also treated with triclosan in solid and liquid cultures at sub-lethal concentrations. Again, we observed serious impairment in colony formation compared to growth in liquid culture (Fig. 5b, c). For *C. glutamicum*, colony-forming activity sharply and completely disappeared when the concentration of triclosan exceeded 2 µg ml^−1^, while the MPN value remained relatively high.

The large reduction in c.f.u. compared to the less sensitive MPN following chemical inhibition with cerulenin and triclosan was suggested to be a specific response, as sub-lethal amounts of ampicillin were not associated with differences between the c.f.u. and MPN values (see Fig. S4).

Based on these results, our findings for *fabB* mutants can not only be extended to wild-type *E. coli*, but also to other bacteria, including *B. subtilis* and *C. glutamicum*: the depletion of fatty acids significantly reduces culturability on solid medium compared to that in liquid medium.

### Fatty acid requirement on the recovery from starvation

**Fig. 6.**
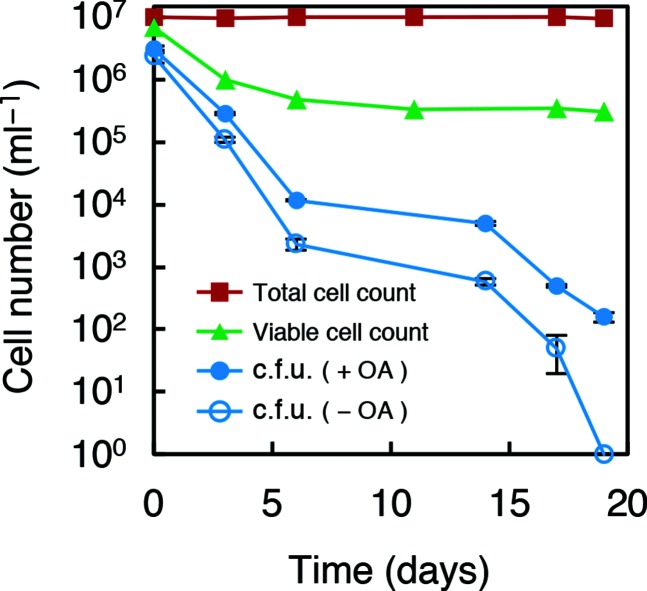
Improved culturability of starved *E. coli* supplemented with unsaturated fatty acids. *E. coli* MG1655 cultures with decreased c.f.u. after exposure to cold and starvation conditions were cultured on M9CAGlc-agarose plates with or without 20 µg ml^−1^ oleic acid (OA). The total cell count and viable cell counts were also determined. The c.f.u. calculation was performed in triplicate, and the error bars indicate standard deviations.

Most bacteria in the natural environment are in a starved state, and only a minor fraction of them form colonies under laboratory conditions. A similar phenomenon is observed in a variety of laboratory strains, including *E. coli*. After prolonged incubation under stressed conditions, such as starvation, *E. coli* cells enter a distinct static state. Such cells subjected to prolonged stresses form few colonies, even on rich medium, despite retaining various signs of viability [51–53]. We predicted that the depletion of fatty acids during this starvation process may be one of the causes of this decrease in colony-forming activity; thus, we examined the effect of fatty acid addition during recovery from stress using M9CAGlc-agarose with or without 20 µg ml^−1^ oleic acid supplementation (Fig. 6). The addition of oleic acid to the plates reduced the decrease in c.f.u. after incubation under stress, elevating colony-forming activity by at least one order of magnitude after 15 days of incubation. This result indicates that starvation stress can cause prototrophic bacteria to partially require fatty acids for colony formation, suggesting that fatty acids are among the limiting factors in colony formation by starved bacteria.

### Fatty acids improve the colony-forming activity of bacteria in soil extracts

**Fig. 7.**
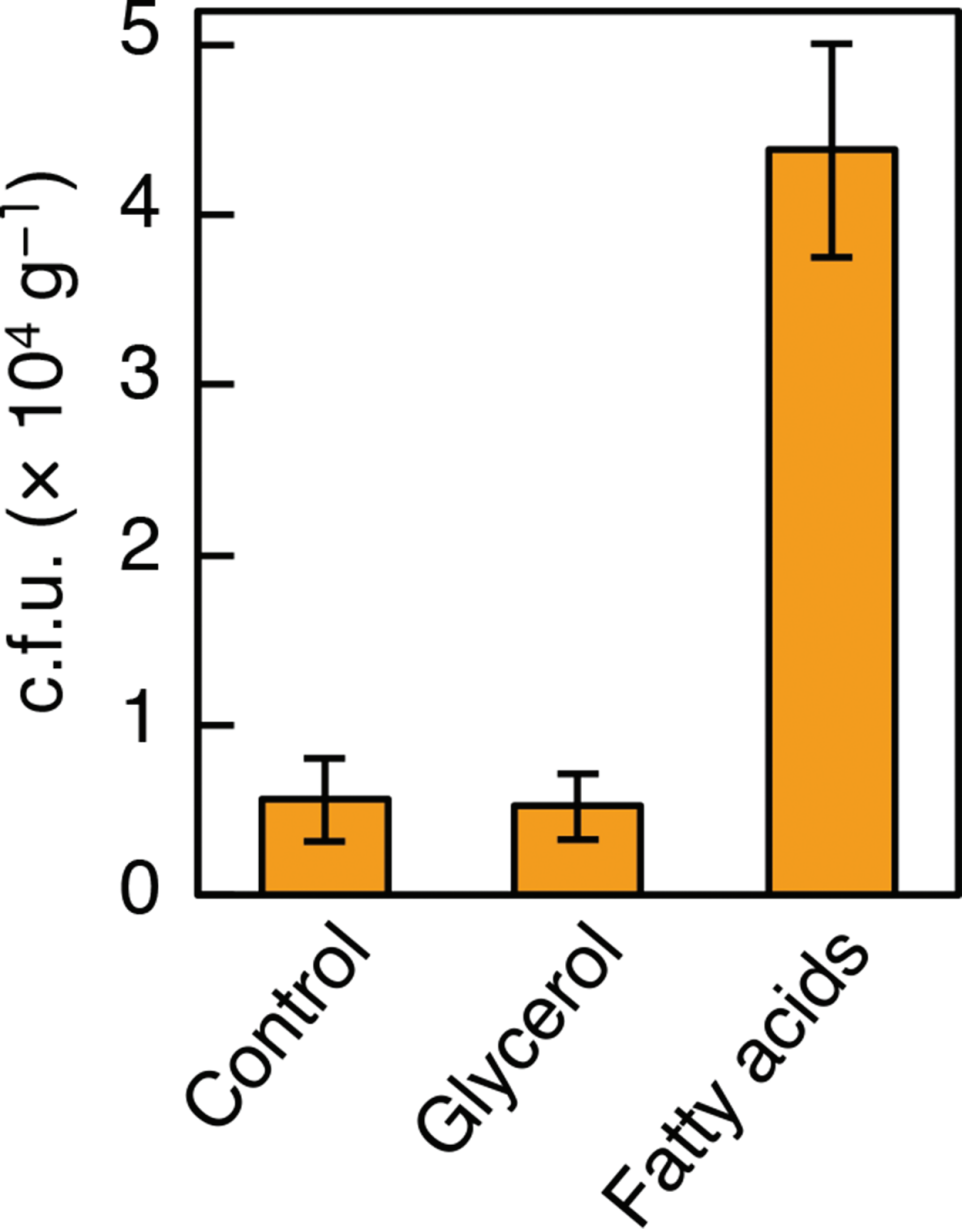
Improved culturability of soil bacteria supplemented with unsaturated fatty acids. The number of bacterial colonies that appeared following the culture of a soil extract on 1/10 strength l-agarose plates with or without 40 µg ml^−1^ fatty acid mixture (oleic and palmitoleic acids) or 100 µg ml^−1^ glycerol after 10 days of incubation at 25 °C. Fifteen plates were used for each condition, and the error bars indicate standard deviations.

The above findings suggested that unsaturated fatty acids are one of the limiting factors in colony formation for starved bacteria in nature. We then examined the effect of fatty acids on the colony-forming activity of soil bacteria. We compared the number of colonies appearing on 1/10 strength l-agarose with and without unsaturated fatty acids (a mixture of 20 µg ml^−1^ each of oleic and palmitoleic acids). The addition of unsaturated fatty acids reproducibly increased the c.f.u. by approximately eightfold (Fig. 7). Fatty acids are not likely to act as a general carbon source, but rather as a stimulatory factor for colony formation, because the quantity of unsaturated fatty acids added was much lower than the quantity of nutrients contained in 1/10 l-broth, and because the addition of 100 µg ml^−1^ glycerol as a control carbon source did not increase colony formation (Fig. 7).

## Discussion

In this study, we aimed to reveal the genetic background limiting bacterial colony formation, and in particular that causing growth differences between solid and liquid culture. To identify the genes responsible for growth differences between solid and liquid media, we first attempted to isolate *E. coli* mutants that could not form colonies but grew in liquid media.

Our screening was based on previous studies of the behaviour of marine bacteria. The liquid-based dilution culturing method generated an order of magnitude more clones than the plating method [32, 33], and thus we expected that most of the clones obtained in liquid culture would not form colonies. However, most clones streaked onto agar plates formed colonies, although the efficiency of colony formation was generally low. This suggests that colony-forming or non-forming behaviour is not a binary trait. Assuming that colony formation is a probabilistic process, we attempted to obtain colonization-defective mutants, as those that showed significantly decreased frequencies of colony formation compared to culturability in liquid medium.

We successfully obtained *E. coli* mutants with decreased colony-forming frequencies relative to growth in liquid culture, first as *fabB*^ts^ mutants and then as the wild-type-based *∆fabB* mutant. Detailed examination of the *fabB* mutants revealed the critical importance of unsaturated fatty acids, particularly for solid culture (Figs 1, 2 and3). Gene *fabB* is unlikely to be the only gene in fatty acid metabolism that is involved in the differences in culturability between solid and liquid media, as we observed a phenotype that was similar to Δ*fabB* in a deletion mutant of *fabA* (T. Mii, K. Nosho and H. Masaki, unpublished data), which encodes 3-hydroxydecanoyl ACP dehydrase in the fatty acid synthesis pathway [54, 55].

The chemical inhibition of fatty acid synthesis with cerulenin or triclosan expanded the results obtained from an *E. coli* mutant to the wild-type strain, and furthermore from *E. coli* to distantly related bacteria, *B. subtilis* and *C. glutamicum.* We showed that sub-lethal concentrations of cerulenin inhibit colony formation to a greater extent than is seen in liquid cultivation, and not only for *E. coli*, but also for *B. subtilis* (Fig. 4). *E. coli* contains homologous condensing enzymes, FabB and FabF, in the elongation cycle of fatty acid synthesis [41]. Cerulenin is known to inhibit FabB strongly, and FabF to a lesser extent [46]. *Bacillus subtilis* does not contain a FabB orthologue and cerulenin acts on its isoform, FabF. In addition, branched-chain fatty acids in *B. subtilis* play a part as unsaturated fatty acids in *E. coli*. Although there are some differences in fatty acid synthases and the types of fatty acids used in *E. coli* and *B. subtilis*, both bacteria showed similar responses to cerulenin. When triclosan was used to inhibit another enzyme involved in fatty acid synthesis, FabI [50], the colony formation of *E. coli* cells was also inhibited compared to growth in liquid culture (Fig. 5a). We observed a similar inhibition of colony formation with triclosan in respect *B. subtilis* and *C. glutamicum* (Fig. 5b, c). In addition, we confirmed that the differences in culturability were caused by the deficit of fatty acids, as the cells were able to form full numbers of colonies following the addition of a small amount of oleic acid (Fig. 5a). Thus, in a wide variety of bacteria, the inhibition of fatty acid synthesis generally shows greater effects on colony formation than during growth in liquid culture. It remains unclear if such chemical inhibitions induce the same metabolic changes as in *E. coli fabB* mutants. However, the large differences between solid and liquid culturability were specific to the inhibition of fatty acid synthesis, as such differences between solid and liquid culturability were not observed when ampicillin was used (Fig. S4).

The results obtained from cells defective in fatty acid synthesis by mutation or the addition of drugs were expanded to wild-type cells under starvation. We observed that starved *E. coli* cells required fatty acids for higher colony formation (Fig. 6), suggesting that depletion of fatty acids is one cause of the low colony-forming frequency in starved bacteria. For further application to bacteria in the natural environment, we showed that the addition of unsaturated fatty acids to a soil extract reproducibly increased the c.f.u. (Fig. 7). This result suggests that depletion of fatty acids may be a limiting factor for colony formation and isolation of bacteria in the natural environment. However, we note that the 100 µg ml^−1^ Triton X-100 we used to disperse fatty acids proved to inhibit colony formation of some bacteria, including *B. subtilis*. Possibly for this reason, the number of colonies per g soil were rather small and the preliminary community analysis of colonies exhibited a population bias to *Proteobacteria* of above 99 %. In spite of such limitations, we observed a modest increase in the genetic diversity of the obtained colonies through fatty acid supplementation (Fig. S5a, b), suggesting that the addition of fatty acids does not impair the diversity of bacteria. We avoided the use of Tween 80 or related ester-type detergents because they could supply fatty acids when bacteria secreted esterases.

In this study, we revealed the critical importance of fatty acids in colony formation in an explicit way by isolating and characterizing *fabB* mutants and showed interesting differences in growth between solid and liquid cultures in wild-type strains under fatty acid depletion conditions. The molecular mechanisms underlying the prominent effect of unsaturated fatty acids remain unclear. It is likely that bacterial cells under starvation conditions require additional fatty acid-associated processes to initiate colony formation on solid culture compared to in liquid culture. Many bacteria in the natural environment may conserve energy by lowering the level of fatty acid synthesis at the expense of colony-forming ability.

Based on the same philosophy as in the present work, in which the low colony-forming state is supposed to be a bacterial genetic response to starvation, we previously took an alternative approach to screen candidates for genetic players in regulating colony formation under the starvation condition [56]. In this line of experiments, we found that cAMP-CRP is critically required for *E. coli* to shift down to a low colony-forming state in response to starvation [56]. Although the different approaches we have taken in the previous study and the present study have not yet determined a common regulating mechanism for colony formation, we believe that our approaches and findings provide a new biological perspective for bacterial growth and contribute to the molecular understanding of the regulation of bacterial colony formation. Furthermore, we expect that our present findings concerning the important role of fatty acids in colony formation will open a way to broaden the accessible bacterial world.

## Supplementary Data

Supplementary File 1
