# Supplementary material for: Isolation of colonization-defective Escherichia coli mutants reveals critical requirement for fatty acids in bacterial colony formation
Source: Microbiology (Reading). 2018 Jul 20;164(9):1122–32. doi: 10.1099/mic.0.000673 (PMC6230765; doi:10.1099/mic.0.000673)
Supplement: Supplementary File 1 [file mic-164-1122-s001.pdf]

Supplementary Material

for “Isolation of colonization-defective *Escherichia coli* mutants reveals critical requirement for fatty acids in bacterial colony formation.” by Nosho *et al*.

This PDF file includes Supplementary methods, Figs. S1–S5 and References.

Supplementary Methods

16S rRNA analysis of formed-colonies of soil bacteria. After incubation of soil bacteria and enumeration of colonies formed on 1/10 strength L-broth solidified with 1.5% Agarose L03 (Fig. 7), genomes were extracted from a few hundred randomly picked colonies from the plates with or without fatty acid mixture, or directly from 4 g of soil using an UltraClean Microbial DNA Isolation Kit (MO BIO Laboratories, Inc., Qiagen N.V., Venlo, Netherlands) according to the manufacturer’s recommendations.

Purified metagenomic DNA was used as a template for generating a 16S rRNA metagenome library. The oligonucleotide primers used for this experiment were 5′-TCGTCGGCAGCGTCAGATGTGTATAAGAGACAGCCTACGGGNGGCWGCAG-3′ and 5′-GTCTCGTGGGCTCGGAGATGTGTATAAGAGACAG GGACTACHVGGGTWTCTAAT-3′, where the underlined regions are the Illumina adapter overhang nucleotide sequences, while the non-underline sequences are locus-specific sequences targeting conserved regions within the V3 and V4 domains of prokaryotic 16S rRNA genes. The locus-specific target sequences were designed based on a reported primer pair, S-D-Bact-0341-b-S-17 and S-D-Bact-0785-a-A-21 [1].

The first PCRs were performed in a 25-μL reaction volume containing 12.5 μL of KAPA HIFI HotStart Ready Mix (KAPA Biosystems, Inc., Wilmington, MA, USA), 1 μM forward and reverse primers, and 12.5 ng of template DNA. Thermal cycling consisted of initial denaturation at 95 °C for 5 min, followed by 25 cycles of denaturation at 95 °C for 30 s, annealing at 55 °C for 30 s, and elongation at 72 °C for 30 s, followed by 72 °C for 5 min. PCR products were purified with an AMPURE XP (Beckman Coulter). Index PCR was conducted in a 50-μL volume containing 25 μL KAPA HIFI HotStart Ready Mix, 5 μL Nextera XT Index Primer 1 (Illumina), 5 μL Nextera XT Index Primer 2 (Illumina), 10 μL sterilized H_2O, and 5 μL first-PCR products. PCR products were purified with an AMPURE XP (Beckman Coulter). Library quality was assessed on an Agilent 2200 Tapestation (Agilent Technologies, Inc.). The libraries were sequenced as paired-end, 300-bp reads using an MiSeq (Illumina) following the manufacturer instructions. All sequence data associated with this project have been submitted to the DNA Data Bank of Japan Sequence Reads Archive (DRA) (accession number: DRA006374).

The overall quality of the MiSeq reads was evaluated using FastQC. The reads were cleaned using the FASTX Toolkit (http://hannonlab.cshl.edu/fastx_toolkit/). The cleaned reads were assembled using FLASH software [2]. Primer sequences were removed from the assembled reads, with average quality <30 and rarefied to 200,000 randomly selected reads per sample to correct for differences in sequencing depth.

Bioinformatics analysis of the assembled reads was performed with QIIME (Quantitative Insights into Microbial Ecology) software, version 1.9.0 [3]. Chimeric reads were detected with ChimeraSlayer against the SILVA 16S rRNA database ver.123 and

removed. Operational taxonomic units (OTUs) were clustered (open-reference OTU "picking" against the SILVA 16S rRNA database ver.123 [4] at 97% similarity using UCLUST [5], and then singleton sequences were removed. Taxonomic assignment of OTUs was then performed using UCLUST against the SILVA 16S rRNA database ver.123. After examining read counts, rarefactions of the OTU table were performed to a chosen maximum subsampling depth of 179,395 sequences and rarefaction curves were plotted. Summary of taxonomic assignments were plotted as bar charts using QIIME. Observed species richness (OTUs number observed) was recorded and compared at a 179,395 sequence depth.

Alpha diversity measurement (Shannon diversity index) was computed using QIIME for all groups [6].

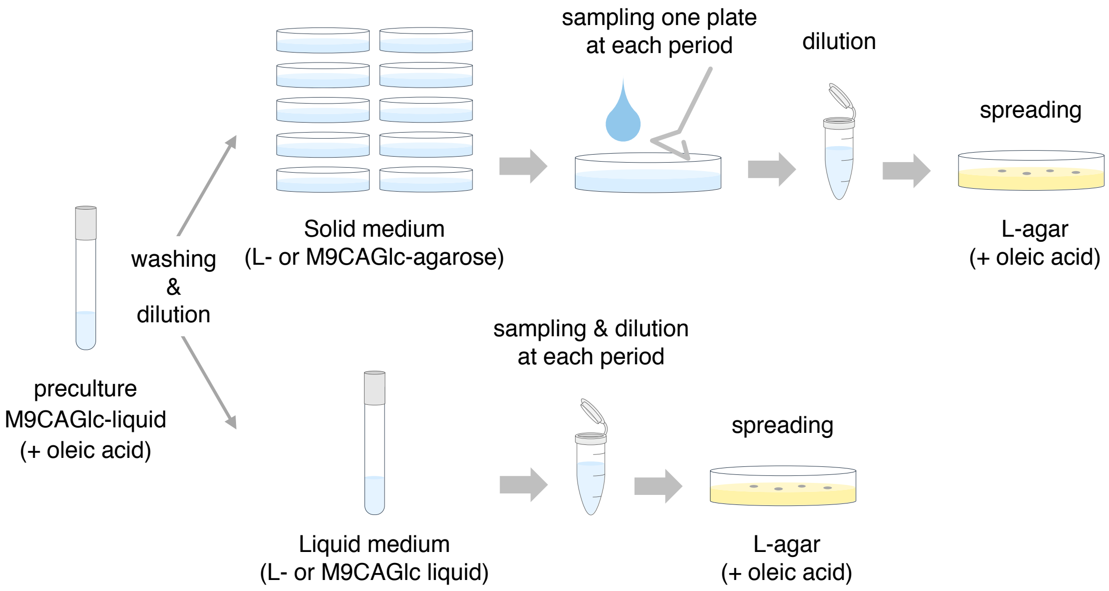

Fig. S1. Procedure for quantifying the number of cells capable of multiplying in solid and liquid media. Washed and diluted pre-cultures were inoculated onto eight plates of solid media and into 10 ml of liquid medium, respectively, as described in the Methods section. At each sampling time, one of the incubated plates was chosen, the cells on the plate were collected, and c.f.u. was counted on L-agar plates containing 20 μg ml^{-1} oleic acid. Similarly, samples were collected from the liquid medium and subjected to c.f.u. counting.

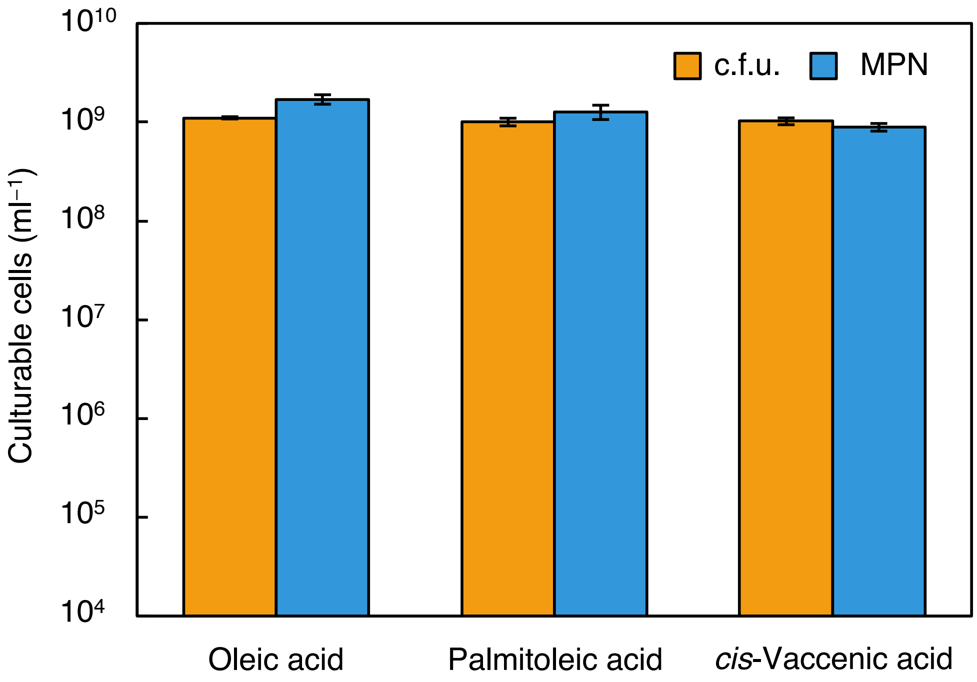

Fig. S2. Culturability of *E. coli* strain Δ*fabB* in solid and liquid cultures supplemented with various unsaturated fatty acids. The c.f.u. and MPN of strain Δ*fabB* were determined in L-agar and liquid L-broth, respectively, after incubation for 4 days at 37 °C with 20 μg ml^{-1} oleic acid, palmitoleic acid, or *cis*-vaccenic acid. Each experiment was performed in triplicate, and the error bars indicate standard deviations.

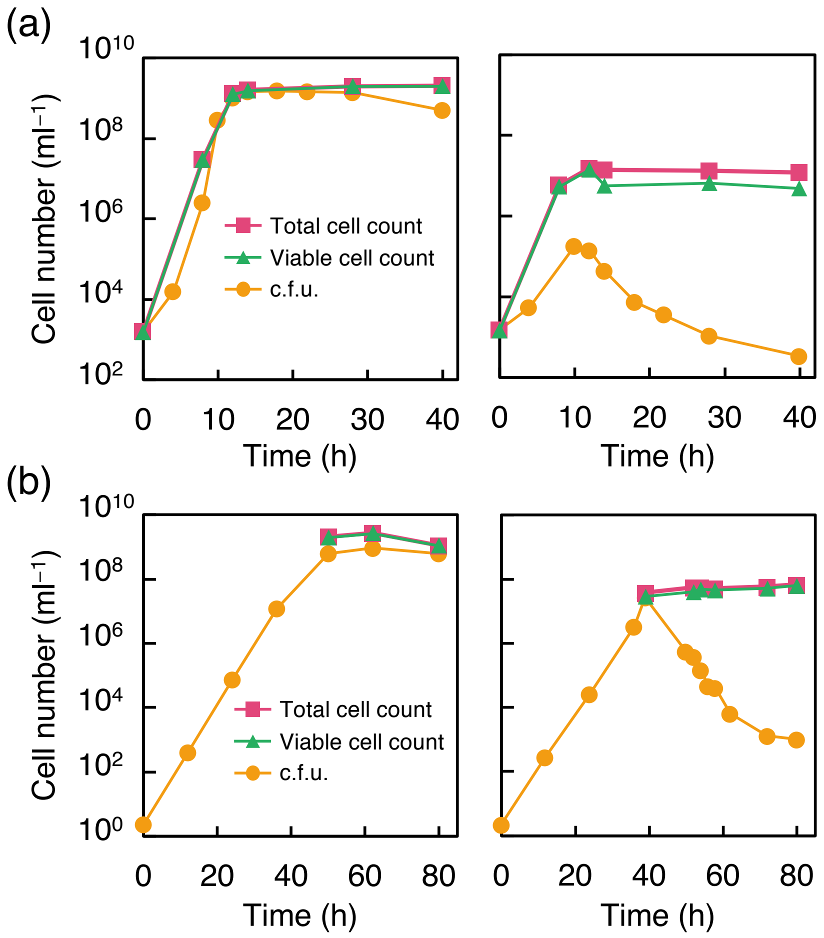

Fig. S3. Viability of strain Δ*fabB* in liquid culture. (a) Total cell count, viable cell count, and c.f.u. of strain Δ*fabB* in L-broth liquid culture with (left) or without (right) 20 μg ml^{-1} of oleic acid. (b) Total cell count, viable cell count, and c.f.u. of strain Δ*fabB* in M9CAGlc liquid culture with 20 μg ml^{-1} (left) or 1 μg ml^{-1} of oleic acid (right). Aliquots were taken at different intervals, and the total cell count was determined with a Coulter counter, viable cell count was calculated by LIVE/DEAD staining, and c.f.u. values were obtained by cultivation on L-agar containing 20 μg ml^{-1} oleic acid.

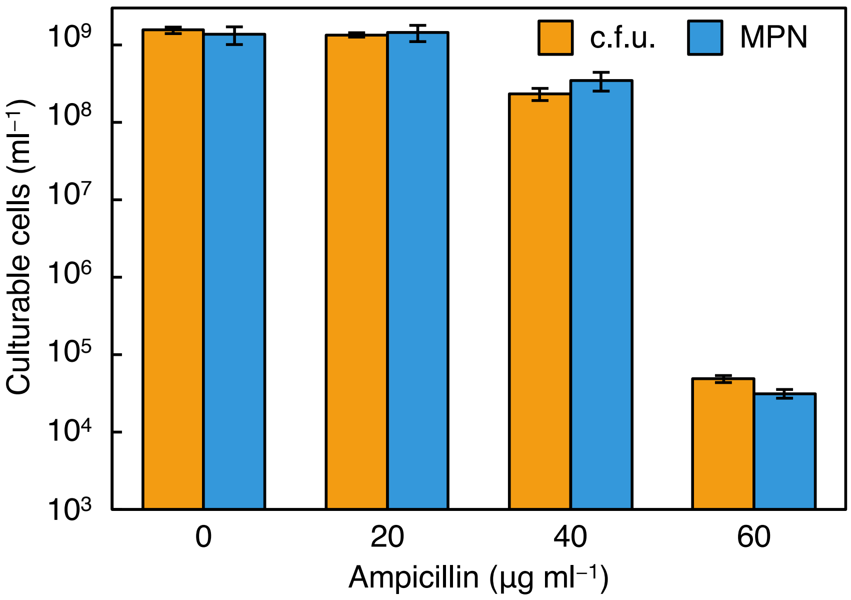

Fig. S4. Comparison of solid and liquid culturability of *E. coli* under ampicillin treatment. The c.f.u. and MPN of *E. coli* MG1655 were determined after incubation for 4 days at 37 °C in M9CAGlc-based solid and liquid media containing various concentrations of ampicillin as indicated. Each experiment was performed in triplicate, and the error bars indicate standard deviations.

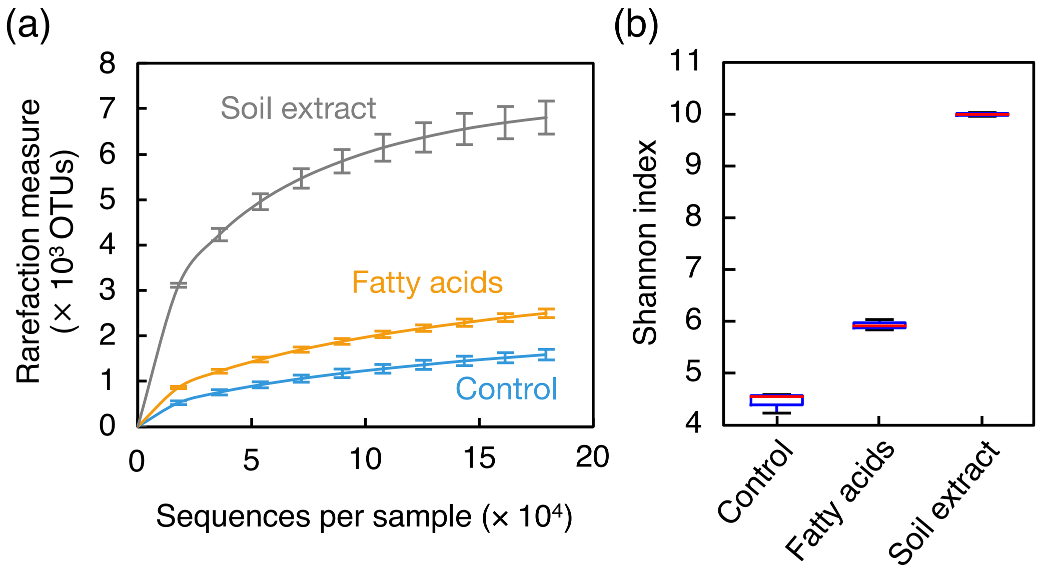

Fig. S5. Improved culturability of soil bacteria supplemented with unsaturated fatty acids. Rarefaction measure (a) and Shannon index (b) from 16S rRNA analysis of randomly picked few hundreds of colonies on 1/10 strength L-agarose plates with or without fatty acid mixture (n = 3) or directly extracted genomes from soil (n = 2).

References

1. **Klindworth A, Pruesse E, Schweer T, Peplies J, Quast C et al.** Evaluation of general 16S ribosomal RNA gene PCR primers for classical and next-generation sequencing-based diversity studies. *Nucleic Acids Res* 2013;41(1):e1.
2. **Magoč T, Salzberg SL**. FLASH: fast length adjustment of short reads to improve genome assemblies. *Bioinformatics* 2011;27(21):2957-2963.
3. **Caporaso JG, Kuczynski J, Stombaugh J, Bittinger K, Bushman FD et al.** QIIME allows analysis of high-throughput community sequencing data. *Nat Methods* 2010;7(5):335-336.
4. **Quast C, Pruesse E, Yilmaz P, Gerken J, Schweer T et al.** The SILVA ribosomal RNA gene database project: improved data processing and web-based tools. *Nucleic Acids Res* 2013;41(D1):D590-D596.
5. **Edgar RC**. Search and clustering orders of magnitude faster than BLAST. *Bioinformatics* 2010;26(19):2460-2461.
6. **Shannon C, Weaver W**. *The mathematical theory of communication*. Urbana: University of Illinois Press; 1949.
